# PET/CT、骨髓活检及骨髓涂片评估弥漫大B细胞淋巴瘤骨髓侵犯的应用及预后价值

**DOI:** 10.3760/cma.j.issn.0253-2727.2022.08.008

**Published:** 2022-08

**Authors:** 显勇 蒋, 东梅 邹, 迎强 张, 孜 王, 薇 张, 道斌 周, 炎 张

**Affiliations:** 1 中国医学科学院、北京协和医学院北京协和医院血液内科，北京 100730 Department of Hematology, Peking Union Medical College Hospital, Chinese Academy of Medical Sciences and Peking Union Medical College, Beijing 100730, China; 2 中国医学科学院、北京协和医学院北京协和医院核医学科，北京 100730 Department of Nuclear Medicine, Peking Union Medical College Hospital, Chinese Academy of Medical Sciences and Peking Union Medical College, Beijing 100730, China

**Keywords:** 淋巴瘤，大B细胞，弥漫性, 骨髓活检, 骨髓涂片, 正电子发射断层显像计算机体层摄影术, Lymphoma, large B-cell, diffuse, Bone marrow biopsy, Bone marrow aspirate, Positron emission tomography combined with computed tomography

## Abstract

**目的:**

探索PET/CT、骨髓活检（BMB）、骨髓涂片（BMA）在评估弥漫大B细胞淋巴瘤（DLBCL）骨髓侵犯中的作用及预后价值。

**方法:**

回顾性分析2015年1月至2017年11月于北京协和医院确诊，且同时具有PET/CT、BMB、BMA结果的DLBCL患者的临床资料。髂骨骨髓SUVmax与正常肝脏组织SUVmax比值≥1为诊断PET/CT骨髓受累的标准。

**结果:**

共纳入76例患者，男32例，中位年龄53（17～79）岁。PET/CT阳性者16例，BMB阳性者12例，BMA阳性者13例。BMB与BMA在诊断DLBCL骨髓侵犯方面具有良好一致性（*κ*＝0.943），PET/CT与BMB（*κ*＝0.763）及BMA（*κ*＝0.776）的一致性略差。中位随访52（0～82）个月，BMB及BMA阳性是总生存的不良预后因素（*P*值分别为0.037和0.007），PET/CT阳性对预后无明显影响（*P*值均>0.05）。

**结论:**

PET/CT、BMB、BMA对于诊断DLBCL骨髓侵犯具有较好的一致性，均为有效的检测手段。BMB和BMA的预后判断价值优于PET/CT。

## Abstract (en)

**Objective:**

This study aimed to determine the value of bone marrow biopsy（BMB）, bone marrow aspiration（BMA）, and positron emission tomography combined with computed tomography（PET/CT）in bone marrow（BM）involvement and prognosis evaluation in diffuse large B-cell lymphoma（DLBCL）.

**Methods:**

The clinical data of patients with DLBCL who underwent PET/CT, BMB, and BMA of the iliac crest were retrospectively analyzed in Peking Union Medical College Hospital from January 2015 to November 2017. The BM involvement on PET/CT was defined as the ratio of maximal standardized uptake values of iliac crest BM to liver parenchyma intensity ≥1.

**Results:**

A total of 76 patients without liver involvement were enrolled, there were 32 males, and the median age was 53（17-79）. Moreover, 16 patients（21.1％）had BM involvement on PET/CT, 12（15.8％）had positive BMB, and 13（17.1％）had positive BMA. Excellent correlation between BMA and BMB（*κ*＝0.943）was found, including good correlation between PET/CT and BMB/BMA（*κ*＝0.763 and 0.776, respectively）. After a median follow-up of 52（0-82）months, BM involvement by BMB（*P*＝0.037）and BMA（*P*＝0.007）were poor prognostic factors for overall survival, positive PET/CT had no significant effect on prognosis（*P*>0.05）.

**Conclusion:**

PET/CT, BMB, and BMA are effective methods to detect BM involvement with great concordance. However, iliac crest BMB and BMA showed superior performance in prognosis evaluation.

弥漫大B细胞淋巴瘤（diffuse large B cell lymphoma，DLBCL）是最常见的侵袭性非霍奇金淋巴瘤（non-Hodgkin's lymphoma，NHL）[1]，高达30％的病例出现骨髓侵犯[2]。骨髓侵犯是预后不良的重要危险因素，也是中枢神经系统复发的高危因素。因此明确有无骨髓侵犯对于DLBCL分期及预后评估等有重要意义[3]。于髂后上棘行骨髓活检（bone marrow biopsy，BMB）是诊断骨髓受累的金标准，但此操作因肿瘤局灶性分布或术者取材不佳可能出现假阴性结果[4]–[5]。正电子发射断层显像计算机体层摄影术（positron emission tomography combined with computed tomography，PET/CT）在DLBCL分期、疗效评估中的价值受到越来越多的重视。对于PET/CT显示骨髓受累的患者，即使未行BMB，其精准分期、治疗选择并不受影响。自2014年Lugano标准提出后，PET/CT已成为DLBCL分期、疗效评价的首选检测方法 [3]–[4],[6]–[7]。骨髓涂片（bone marrow aspirate，BMA）作为一种经典的、广泛采用的检测方法，可细致观察骨髓细胞形态并发现异常淋巴细胞，也是淋巴瘤骨髓侵犯的有效检测方法。本中心既往研究显示，BMA对诊断DLBCL骨髓侵犯具有良好的敏感性和特异性[8]。

既往鲜有研究对上述三种检测方法的一致性进行探索。本研究通过回顾性分析本中心DLBCL患者PET/CT及骨髓检查结果，评价BMB、BMA及PET/CT诊断DLBCL骨髓侵犯的一致性，同时探索不同检测方法的预后价值。

## 病例与方法

1. 病例：纳入2015年1月至2017年11月就诊于北京协和医院血液内科，病理明确诊断为DLBCL的患者，所有患者初始治疗前完成^18^F-氟代脱氧葡萄糖（FDG）PET/CT、BMB及BMA。本研究需要通过肝脏标准摄取值（standard uptake value，SUV）与髂骨SUV进行比较判断骨髓是否受累，故基线PET/CT显示肝脏受累的患者未纳入。

2. PET/CT图像采集及分析：PET/CT仪器型号为Siemens Biograph Truepoint TrueV，显像剂为^18^F-FDG（剂量0.15 mCi/kg，约5.55 MBq/kg，静脉注射），标记率大于98％。注射前，患者需禁食>6 h，控制血糖≤10 mmol/L。注射后，患者安静休息50 min，排尿，行常规PET/CT扫描。先后采集颈部至下肢低剂量CT（35 mA，120 kV）、下肢至颈部的PET图像（2 min/床位，5个床位），然后将图像进行匹配、融合。根据前期研究结果[8]，采用SUVmax比值法，即髂后上棘SUVmax/正常肝脏SUVmax（简称SUVmax比值）≥1作为判断PET-CT阳性的标准。

3. BMA与BMB：选择单侧髂后上棘采集标本进行BMA及BMB。对BMA进行瑞氏-吉姆萨染色，观察细胞形态并分类，200个有核细胞中异常淋巴细胞数/总有核细胞数≥1％诊断为淋巴瘤骨髓侵犯[9]。对活检骨髓组织进行甲醛固定、石蜡包埋及切片，HE染色，观察细胞形态。BMB结合免疫组化染色判断有无淋巴瘤骨髓侵犯[10]。所有检查结果均经2名有资质的影像学、病理或形态学医师判读，不向影像学医师提供患者其他临床及病理资料，采用盲法阅片以减少偏倚。

4. 随访：采用查阅患者门诊、住院病历的形式进行随访。总生存（OS）期定义为自DLBCL开始治疗至因任何原因死亡或末次随访时间，无进展生存（PFS）期定义为自DLBCL开始治疗至疾病首次复发、进展、因任何原因死亡或末次随访的时间。

5. 统计学处理：用SPSS 25.0软件进行统计学分析，Kappa检验检测BMB、BMA及PET/CT在DLBCL骨髓侵犯诊断上的一致性，*κ*>0.75则认为2种方法具有良好一致性。计数资料用例数（百分比）表示，计量资料用中位数（范围）或均数±标准差表示。以*t*检验评估组间差异，双侧*P*<0.05为差异有统计学意义。采用Kaplan-Meier法计算OS及PFS，采用Log-rank检验比较组间生存差异，采用Cox比例风险回归模型进行多因素分析。

## 结果

1. 基本资料：76例患者中，男32例，女44例，中位年龄53（17～79）岁，≤60岁者55例（72.4％）。75例（98.7％）为初诊患者。Ann Arbor分期以Ⅳ期为主，51例（67.1％），Ⅰ、Ⅱ、Ⅲ期分别为5例（6.6％）、11例（14.5％）、9例（11.8％）。国际预后指数（IPI）0～1分（低危）22例（28.9％），2分（中低危）14例（18.4％），3分（中高危）24例（31.6％），4～5分16例（21.1％）。根据Hans分类方法，非生发中心亚型45例（59.2％），生发中心亚型24例（31.6％），7例（9.2％）无法分类。8例（10.5％）患者BMA可见吞噬血细胞现象。

2. BMB与BMA的一致性分析：76例DLBCL患者中，14例通过BMA或BMB诊断为骨髓侵犯，其中13例BMA阳性，12例BMB阳性，两者均阳性者11例。Kappa一致性检验显示，*κ*＝0.943，提示BMA和BMB在诊断DLBCL骨髓侵犯上具有良好一致性。相对于BMB，BMA检测骨髓侵犯的敏感性为91.7％（11/12），特异性为96.9％（62/64），阳性预测值为84.6％（11/13），阴性预测值为98.4％（62/63）。

3. PET/CT与BMB、BMA的一致性分析：76例DLBCL患者中PET/CT阳性者16例，BMB阳性者12例，BMA阳性者13例。PET/CT及BMB同时阳性者5例，PET/CT阳性而BMB阴性11例，PET/CT阴性而BMB阳性7例。Kappa一致性检验显示，PET/CT与BMB的*κ*值为0.763，提示PET/CT与BMB在诊断DLBCL骨髓侵犯上具有良好的一致性。相对于BMB，PET/CT检测骨髓侵犯的敏感性为41.6％（5/12），特异性为82.8％（53/64），阳性预测值为31.3％（5/16），阴性预测值为88.3％（53/60）。PET/CT与BMA的*κ*值为0.776，提示PET/CT与BMA在诊断DLBCL骨髓侵犯中同样具有良好一致性。

4. BMB、BMA与PET/CT髂骨SUVmax、SUVmax比值的相关性：BMB阳性组（12例）PET/CT髂骨SUVmax为4.82±6.80，SUVmax比值为1.89±3.33。BMB阴性组（64例）PET/CT髂骨SUVmax为2.33±2.52，SUVmax比值为0.81±0.70。BMB阳性组与阴性组PET/CT髂骨SUVmax（*P*＝0.169）、SUVmax比值（*P*＝0.215）的差异均无统计学意义。

BMA阳性组PET/CT髂骨SUVmax高于BMA阴性组（4.89±2.09对2.14±0.24，*P*＝0.012）。根据BMA中淋巴瘤细胞比例是否大于10％将BMA阳性者（13例）分为两组，其中>10％组7例，≤10％组6例，淋巴瘤细胞>10％组PET/CT髂骨SUVmax更高，但组间差异无统计学意义（7.63±4.40对2.55±0.61，*P*＝0.241）。

5. 三种骨髓受累评估方式对预后判断的价值：随访截止时间为2021年11月，76例患者中，71例获得生存随访数据。中位随访52（0～82）个月，2年PFS率和OS率分别为83.3％和89.3％。其中BMB阳性、BMA阳性是OS的不良预后因素（*P*值分别为0.037和0.007）（图1、图2），对PFS无明显影响（*P*值均>0.1）。PET/CT是否阳性对PFS、OS无明显影响（*P*值均>0.05）。

**图1:**
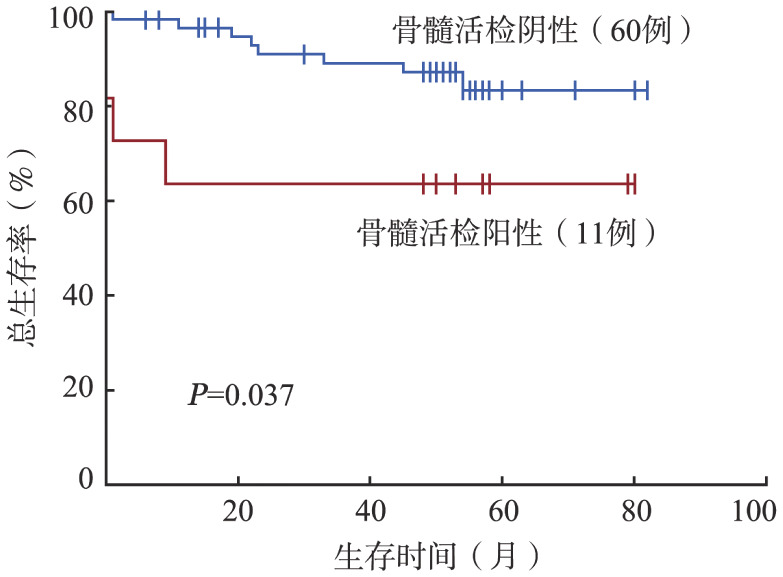
骨髓活检阳性组与阴性组患者的总生存曲线

**图2:**
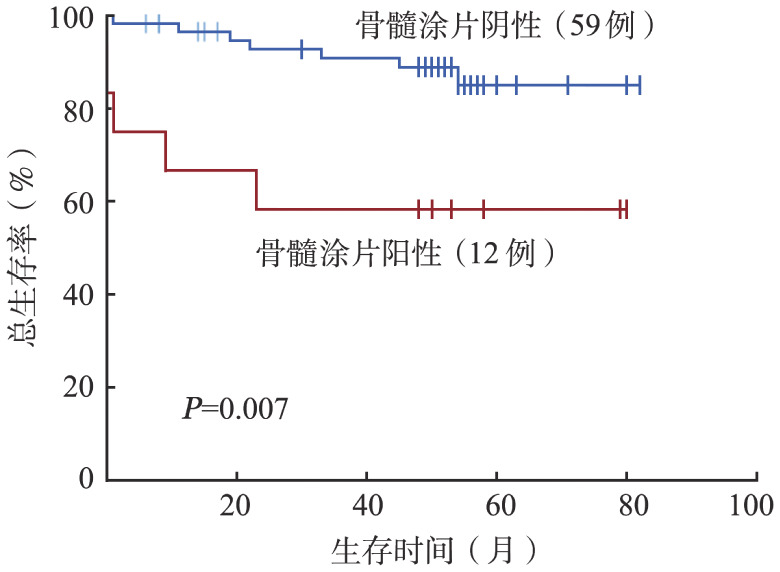
骨髓涂片阳性组与阴性组患者的总生存曲线

同时，本研究7例患者基线PET/CT提示髂骨外病灶存在（表1），化疗后上述高代谢病灶消失，此种情况也可认为存在骨髓受累。将上述7例患者与SUVmax比值法诊断的16例PET/CT阳性患者合并，共23例患者存在PET/CT提示的骨髓受累。Log-rank检验提示，PET/CT骨髓受累组（23例）与PET/CT无骨髓受累组（48例）PFS及OS的差异均无统计学意义（*P*值均>0.1）。Cox回归分析显示，BMB阳性与BMA阳性是OS的不良预后因素（*P*值分别为0.050和0.013），PET/CT提示髂骨或髂骨外病灶不是PFS和OS的预后因素（*P*值均>0.1）（表2）。

**表1:** 7例PET/CT提示髂骨外骨髓病灶的弥漫大B细胞淋巴瘤患者的临床资料

例号	性别	年龄（岁）	受累部位	病灶SUVmax	SUVmax比值（髂骨/肝脏）	髂骨骨髓涂片	髂骨骨髓活检
1	女	42	股骨	2.16	0.55	阴性	阴性
2	女	37	脊柱、胸骨	不详	0.76	阴性	阴性
3	女	68	胸腰椎、肋骨、左髂骨	4.01	0.54	阴性	阴性
4	女	60	腰椎	不详	0.94	阴性	阴性
5	女	70	胸椎	不详	0.79	阳性	阴性
6	女	73	肱骨、锁骨	12.8	0.42	阴性	阴性
7	女	39	颈胸椎、肩胛骨、肋骨	4.47	0.56	阴性	阴性

**表2:** Cox回归分析PET/CT、骨髓活检、骨髓涂片阳性对弥漫大B细胞淋巴瘤患者预后的影响

因素	无进展生存		总生存	
	*P*值	*HR*（95％ *CI*）	*P*值	*HR*（95％ *CI*）
骨髓活检（阳性，阴性）	0.352	1.834（0.511～6.582）	0.050	3.321（0.998～11.050）
骨髓涂片（阳性，阴性）	0.122	2.503（0.783～7.995）	0.013	4.299（1.362～13.567）
髂骨PET/CT（阳性，阴性）	0.522	1.630（0.365～7.288）	0.315	2.855（0.368～22.118）
髂骨或髂骨外PET/CT（阳性，阴性）	0.437	1.659（0.462～5.953）	0.296	2.250（0.492～10.283）

## 讨论

本研究评估了BMA、BMB及PET/CT诊断DLBCL骨髓侵犯的一致性，并比较了三者的预后评估价值。目前多种检测方法用于评估骨髓侵犯，包括BMB、BMA、骨髓流式细胞术分析、PET/CT等。BMB是诊断DLBCL骨髓侵犯的金标准。虽然骨髓液标本进行流式细胞术分析在理论上具有更好的敏感性和特异性，但在临床工作中对骨髓受累的检出并未体现出优势。2021年一项研究比较了BMB与流式细胞术检测DLBCL骨髓受累的优劣，结果显示阳性率分别为7.7％和15.4％，一致性为85.5％[11]。但限于流式细胞术对设备要求较高，并非所有中心均将流式细胞术作为初始评估项目，国内外指南亦未将骨髓流式细胞术列入一线推荐。BMA可以充分展示骨髓细胞形态，操作便捷，成本低廉。我们前期工作证实BMB和BMA具有较好的一致性[8]。本研究对BMA及BMB的一致性再次进行验证，*κ*值为0.946，提示两者在DLBCL骨髓侵犯的诊断中具有极好的一致性，与既往其他研究结果类似[12]–[13]。BMA也存在局限性，与BMB及骨髓流式细胞术检查相比，BMA判读的主观性更大，对阅片人的经验和责任心要求更高。但联合上述检查可获得更高的敏感性和特异性，Okamoto等[11]报道，在184例DLBCL患者中，BMA的骨髓受累检出率为15.4％，联合骨髓流式细胞术可将检出率提升到22.6％。

2010年以来，多项研究证实PET/CT在诊断骨髓受累方面与BMB具有良好的一致性[4],[6],[14]–[16]。2017年Teagle等[6]报道，以BMB结果作为参照，PET/CT诊断DLBCL骨髓侵犯的敏感性及特异性均为100％，但实际操作中仍有较多变数。

PET/CT骨髓受累的评判标准尚未统一。文献报道中用于评判骨髓受累的方法包括：骨髓局灶性摄取SUVmax≥2.5；视觉比较法，即骨髓摄取值高于肝脏组织；SUV比值法，即骨髓SUVmax与正常肝脏SUVmax比值≥1；治疗后高摄取病灶消失等。本研究采用的髂骨/肝脏SUVmax比值法基于本中心前期研究结果。在本研究中再次验证PET/CT及BMB具有良好一致性（*κ*＝0.790），提示髂骨/肝脏SUVmax比值法是一种有效的评估骨髓受累的方法。需关注的是，本研究中有18例患者PET/CT与BMB结果不一致，分别为PET/CT阳性、BMB阴性患者11例；PET/CT阴性、BMB阳性患者7例。其中7例BMB阳性患者的病理均显示散在肿瘤细胞浸润，肿瘤细胞异形性较明显，并非惰性B淋巴细胞侵犯骨髓，髂骨SUVmax最高值为3.37。上述结果提示，淋巴瘤细胞浸润的方式与SUV升高程度相关，散在肿瘤细胞浸润相关的SUV异常主要表现为轻度升高，而骨髓中肿瘤细胞大量聚集形成瘤体时，可表现为SUV显著升高。因此骨髓SUV轻度升高的患者应综合应用多种检查方法评估骨髓受累情况。

PET/CT骨髓受累是否可作为预后评估因素亦有争议。2014年Adams等[17]回顾性分析了117例初治DLBCL患者的临床资料，发现PET/CT显示的骨髓受累并不能预测PFS和OS。Cerci等[18]基于327例DLBCL患者的研究发现，PET/CT显示骨骼异常代谢而BMB阴性与预后不良无关，BMB阳性与较短的无病生存期和OS期相关。Vishnu等[19]和Alzahrani等[15]也得到了类似结论，虽然PET/CT在检测骨髓受累方面具有很高的阴性预测值，但未能预测不良预后。而El Karak等[20]发现，PET/CT对DLBCL骨髓侵犯的诊断不但敏感性优于BMB，对不良预后的预测价值也优于BMB。本研究首次在国内患者中评估BMB、BMA和PET/CT的预后价值，与既往大多数研究相似，BMB是独立的预后不良因素。因BMA与BMB具有良好的一致性，亦可作为OS的不良预后因素，而PET/CT骨髓受累尚不是PFS和OS的预后因素。

本研究的局限性如下，作为一项小规模单中心回顾性研究，不可避免地存在选择偏倚；病理及影像学医师可能会互相参考对方结果，导致判读偏差等。但本研究通过患者连续入组、影像及病理科医师盲法阅片等方法尽可能减低干扰因素，保证研究的科学性。

本研究的创新之处在于，确认了BMA在诊断DLBCL骨髓受累中的地位，并显示出了优于PET/CT的预后价值。因此，应重视BMA在检测DLBCL骨髓受累中的应用，特别是在未能广泛开展PET/CT或骨髓流式细胞术检查的基层单位。

本研究通过单中心回顾性分析证实PET/CT、BMB、BMA对于诊断DLBCL骨髓侵犯具有较好的一致性，均为有效的检测手段。其中髂后上棘BMB和BMA的预后判断价值优于PET/CT。
